# Supplementary material for: A systematic review and meta-analysis of eyespot anti-predator mechanisms
Source: eLife. 2024 Dec 12;13:RP96338. doi: 10.7554/eLife.96338 (PMC11637465; doi:10.7554/eLife.96338)
Supplement: Supplementary file 2. [file elife-96338-supp2.docx]

**Supplementary file 2a**. Included studies.

| **title** | **year** | **authors** | **journal** | **doi** |
| --- | --- | --- | --- | --- |
| The Function of Eyespot Patterns in the Lepidoptera | 1957 | Blest, AD. | Behaviour | 10.1163/156853956X00048 |
| Reactions of male domestic chicks to two-dimensional eye-like shapes | 1980 | Jones, RB. | Animal Behaviour | 10.1016/S0003-3472(80)80025-X |
| The Feeding Behaviour of Starlings (*Sturnus vulgaris*) in the Presence of ‘Eyes’ | 1983 | Inglis, IR., Huson, LW., Marshall, MB. and Neville, PA. | Zeitschrift für Tierpsychologie | 10.1111/j.1439-0310.1983.tb02151.x |
| Butterfly wing markings are more advantageous during handling than during the initial strike of an avian predator | 1985 | Wourms, MK. and Wasserman, FE. | Evolution | 10.1111/j.1558-5646.1985.tb00426.x |
| Significance of butterfly eyespots as an anti-predator device in ground-based and aerial attacks | 2003 | Lyytinen, A., Brakefieid, PM. and Mappes, J. | Oikos | 10.1034/j.1600-0706.2003.11935.x |
| Does predation maintain eyespot plasticity in *Bicyclus anynana*? | 2004 | Lyytinen, A., Brakefield, PM., Lindström, L., and Mappes, J. | Proceedings of the Royal Society B: Biological Sciences | 10.1098/rspb.2003.2571 |
| Asymmetry in size, shape, and color impairs the protective value of conspicuous color patterns | 2004 | Forsman, A. and Herretröm, J. | Behavioral Ecology | 10.1093/beheco/arg092 |
| Prey survival by predator intimidation: an experimental study of peacock butterfly defence against blue tits | 2005 | Vallin, A, Jakobsson, S., Lind, J. and Wiklund, C. | Proceedings of the Royal Society B: Biological Sciences | 10.1098/rspb.2004.3034 |
| Field experiments on the effectiveness of 'eyespots' as predator deterrents | 2007 | Stevens, M., Hopkins, E., Hinde, W., Adcock, A., Connolly, Y., Troscianko, T. and Cuthill, IC. | Animal Behaviour | 10.1016/j.anbehav.2007.01.031 |
| The anti-predator function of 'eyespots' on camouflaged and conspicuous prey | 2008 | Stevens, M., Stubbins, CL. and Hardman, CJ. | Behavioral Ecology and Sociobiology | 10.1007/s00265-008-0607-3 |
| Conspicuousness, not eye mimicry, makes "eyespots" effective antipredator signals | 2008 | Stevens, M., Hardman, CJ. and Stubbins, CL. | Behavioral Ecology | 10.1093/beheco/arm162 |
| The protective value of conspicuous signals is not impaired by shape, size, or position asymmetry | 2009 | Stevens, M., Castor-Perry, SA. and Price, JRF. | Behavioral Ecology | 10.1093/beheco/arn119 |
| The function of animal 'eyespots': Conspicuousness but not eye mimicry is key | 2009 | Stevens, M., Cantor, A., Graham, J. and Winney, IS. | Current Zoology | 10.1093/czoolo/55.5.319 |
| Fixed eyespot display in a butterfly thwarts attacking birds | 2009 | Kodandaramaiah, U., Vallin, A. and Wiklund, C. | Animal Behaviour | 10.1016/j.anbehav.2009.02.018 |
| Can we use starlings' aversion to eyespots as the basis for a novel 'cognitive bias' task? | 2009 | Brilot, BO., Normandale, CL., Parkin, A. and Bateson, M. | Applied Animal Behaviour Science | 10.1016/j.applanim.2009.02.015 |
| Constant eyespot display as a primary defence-survival of male and female emperor moths attacked by blue tits | 2010 | Vallin, A., Sven J. and Christer W. | The Journal of Research on the Lepidoptera | 10.5962/p.266504 |
| Deflective effect and the effect of prey detectability on anti-predator function of eyespots | 2011 | Vallin, A. and Dimitrova, M., Kodandaramaiah, U. and Merilaita, S. | Behavioral Ecology and Sociobiology | 10.1007/s00265-011-1173-7 |
| Number of eyespots and their intimidating effect on naïve predators in the peacock butterfly | 2011 | Merilaita, S., Vallin, A., Kodandaramaiah, U., Dimitrova, M., Ruuskanen, S. and Laaksonen, T. | Behavioral Ecology | 10.1093/beheco/arr135 |
| The 'sparkle' in fake eyes - the protective effect of mimic eyespots in lepidoptera | 2012 | Blut, C., Wilbrandt, J., Fels, D., Girgel, EI.and Lunau, K. | Entomologia Experimentalis et Applicata | 10.1111/j.1570-7458.2012.01260.x |
| Eyespots interact with body colour to protect caterpillar-like prey from avian predators | 2012 | Hossie, T.J. and Sherratt, T.N. | Animal Behaviour | 10.1016/j.anbehav.2012.04.027 |
| Anti-predator adaptations and strategies in the Lepidoptera | 2012 | de Wert, L. | Doctoral thesis | none |
| Bird attacks on a butterfly with marginal eyespots and the role of prey concealment against the background | 2013 | Olofsson, M., Jakobsson, S. and andWiklund, C, | Biological Journal of the Linnean Society | 10.1111/bij.12063 |
| Defensive posture and eyespots deter avian predators from attacking caterpillar models | 2013 | Hossie, TJ and Sherratt, TN | Animal Behaviour | 10.1016/j.anbehav.2013.05.029 |
| Revealed by conspicuousness: distractive markings reduce camouflage | 2013 | Stevens, M., Marshall, KLA, Troscianko, J., Finlay, S., Burnand, D. and Chadwick, SL. | Behavioral Ecology | 10.1093/beheco/ars156 |
| Eyespot display in the peacock butterfly triggers antipredator behaviors in naïve adult fowl | 2013 | Olofsson, M., Lovlie, H., Tibblin, J., Jakobsson, S. and Wiklund, C. | Behavioral Ecology | 10.1093/beheco/ars167 |
| The position of eyespots and thickened segments influence their protective value to caterpillars | 2014 | Skelhorn, J., Dorrington, G., Hossie, TJ. and Sherratt, TN. | Behavioral Ecology | 10.1093/beheco/aru154 |
| Predator mimicry, not conspicuousness, explains the efficacy of butterfly eyespots | 2015 | De Bona, S., Valkonen, JK., López-Sepulcre, A. and Mappes, J. | Proceedings of the Royal Society B: Biological Sciences | 10.1098/rspb.2015.0202 |
| Body size affects the evolution of eyespots in caterpillars | 2015 | Hossie, TJ., Skelhorn, J., Breinholt, JW., Kawahara, AY. and Sherratt, TN. | Proceedings of the National Academy of Sciences of the United States of America | 10.1073/pnas.1415121112 |
| What makes eyespots intimidating- the importance of pairedness Evolutionary ecology and behaviour | 2015 | Mukherjee, R. and Kodandaramaiah, U. | BMC Evolutionary Biology | 10.1186/s12862-015-0307-3 |
| On the deterring effect of a butterfly's eyespot in juvenile and sub-adult chickens | 2015 | Olofsson, M., Wiklund, C. and Favati, A | Current Zoology | 10.1093/czoolo/61.4.749 |
| Multicomponent deceptive signals reduce the speed at which predators learn that prey are profitable | 2016 | Skelhorn, J., Holmes, GG., Hossie, T.J. and Sherratt, TN. | Behavioral Ecology | 10.1093/beheco/arv135 |
| Attack risk for butterflies changes with eyespot number and size | 2016 | Ho, S., Schachat, SR., Piel, WH. and Monteiro, A. | Royal Society Open Science | 10.1098/rsos.150614 |
| The effectiveness of eyespots and masquerade in protecting artificial prey across ontogenetic and seasonal shifts | 2022 | Postema, EG. | Current Zoology | 10.1093/cz/zoab082 |

**Supplementary file 2b**. Excluded studies.

| **title** | **year** | **authors** | **journal** | **doi** | **reason** |
| --- | --- | --- | --- | --- | --- |
| The effects of a tranquilliser on the reactions of domestic chicks to an aversive eye-like shape | 1979 | Jones, RB. | IRCS Medical Science | none | No full-text |
| Young domestic chicks avoid eye-like shapes | 1980 | JONES, RB | Applied Animal Ethology | 10.1016/0304-3762(80)90037-1 | No full-text |
| The startle responses of blue jays to Catocala (*Lepidoptera: Noctuidae*) prey models | 1985 | Schlenoff, DH. | Animal Behaviour | 10.1016/S0003-3472(85)80164-0 | Wrong outcome |
| Fearful symmetry: Pattern size and asymmetry affects aposematic signal efficacy | 1999 | Forsman, A. and Merilaita, S. | Evolutionary Ecology | 10.1023/A:1006630911975 | Invaild comparator |
| "An eye for an eye?" - On the generality of the intimidating quality of eyespots in a butterfly and a hawkmoth | 2007 | Vallin, A., Jakobsson, S. and Wiklund, C. | Behavioral Ecology and Sociobiology | 10.1007/s00265-007-0374-6 | Invaild comparator |
| Coincident disruptive coloration | 2009 | Cuthill, IC and Szekely, A | Philosophical Transactions of the Royal Society B-Biological Science | 10.1098/rstb.2008.0266 | Invaild comparator |
| Marginal eyespots on butterfly wings deflect bird attacks under low light intensities with UV wavelengths | 2010 | Olofsson, M., Vallin, A., Jakobsson, S. and Wiklund, C. | PLoS ONE | 10.1371/journal.pone.0010798 | Invaild comparator |
| Insect coloration as a defence mechanism against visually hunting predators | 2011 | Lyytinen, A. | Doctoral thesis | none | Published thesis |
| Effects of lepidopteran eyespot components on the deterrence of predatory birds | 2015 | Blut, C. and Lunau, K. | Behaviour | 10.1163/1568539X-00003288 | Invaild comparator |
| Antipredator behavior by a nesting hummingbird in response to a caterpillar with eyespots | 2019 | Marden, JH. and Pérez Carrillo, JF. | Ecology | 10.1002/ecy.2582 | Obseravational study |
| The Influence of the eyespots of peacock butterfly (*Aglais io*) and caterpillar on predator recognition | 2020 | Park, J. and Heo D | Open Science Journal | 10.23954/osj.v5i2.2455 | Invaild comparator |
