# Supplementary material for: A systematic review and meta-analysis of eyespot anti-predator mechanisms
Source: eLife. 2024 Dec 12;13:RP96338. doi: 10.7554/eLife.96338 (PMC11637465; doi:10.7554/eLife.96338)
Supplement: Supplementary file 3. — The bold typeface is used when a 95% confidence interval (CI) does not contain zero; thus, it can be interpreted as an existing significant effect in predator avoidance. [file elife-96338-supp3.docx]

**Supplementary file 3**

|  | Estimate | 95%CI |
| --- | --- | --- |
| intercept | -0.06 | (-0.50, 0.34) |
| Treatment stimulus | -0.02 | (-0.19, 0.23) |
| **Log-transformed area** | **0.09** | **(0.009, 0.18)** |
| Number pattern | -0.05 | (-0.11, 0.004) |
| Material type of prey: real | 0.18 | (-0.09, 0.45) |
