# Supplementary material for: A systematic review and meta-analysis of eyespot anti-predator mechanisms
Source: eLife. 2024 Dec 12;13:RP96338. doi: 10.7554/eLife.96338 (PMC11637465; doi:10.7554/eLife.96338)
Supplement: Supplementary file 4. — We accessed Scopus, ISI Web of Science core collection, Google Scholar (Japanese, Polish, Portuguese, Russian, Spanish, Simplified Chinese, and Traditional Chinese) on 08/06/2023, and Bielefeld Academic Search Engine (BASE) on 26/06/2023. BASE was used as a source of grey literature. We conducted backward and forward reference searches for key review articles using Scopus on 19/06/2023. We modified search strings to collect studies to capture studies examining the effects of eyespot patterns on birds using experimental methods. Search strings were adapted to the structure of each database. [file elife-96338-supp4.docx]

**Supplementary file 4**

| Database | Search strings |
| --- | --- |
| Scopus | TITLE-ABS-KEY ( ( ( eyespot* OR eye-spot* OR "eye spot*" OR eye-like* OR "eye like*" OR eye-mimic* OR "eye mimic*" OR "eye similari*" OR "predator* eye*" OR "eye similar*" OR concentric*) AND ( attack* OR antipredator* OR anti-predator* OR aposematic* OR avoid* OR conspicuous* OR warn* OR fear* OR intimidat* OR predator-prey* OR butterfl* OR moth* OR bird* OR avian* OR caterpillar* OR prevent* OR aves OR passeri*) ) AND NOT ( fish* OR manti* OR lizard* OR bat* OR nano* OR health* OR patients OR women OR men OR children OR pediatric OR medic* OR hormon* OR genes OR magnet* OR valve* OR fluid* OR concrete OR beam* OR tissue* OR charge* OR energ* OR electro* ) ) |
| ISI Web of Science | TS = ( ( ( eyespot* OR eye-spot* OR "eye spot*" OR eye-like* OR "eye like*" OR eye-mimic* OR "eye mimic*" OR "eye similari*" OR "predator* eye*" OR "eye similar*" OR concentric*) AND ( attack* OR antipredator* OR anti-predator* OR aposematic* OR avoid* OR conspicuous* OR warn* OR fear* OR intimidat* OR predator-prey* OR butterfl* OR moth* OR bird* OR avian* OR caterpillar* OR prevent* OR aves OR passeri*) ) NOT ( fish* OR manti* OR lizard* OR bat* OR nano* OR health* OR patients OR women OR men OR children OR pediatric OR medic* OR hormon* OR genes OR magnet* OR valve* OR fluid* OR concrete OR beam* OR tissue* OR charge* OR energ* OR electro* ) ) |
| BASE | eyespot* AND (avoid* predator* prevent* intimidat* mimi*) AND (ave* bird* passerine* butterfl* moth* lepidoptera caterpillar*) AND (experiment* stud*) |
| Google scholar | eyespot avoid\|predator\|prevention\|intimidation\|mimic aves\|bird\|passerine\|butterfly\|moth\|lepidoptera\|caterpillar experiment\|study  We translated the above English search string into *Japanese*, *Polish*, *Portuguese*, *Russian*, *Spanish*, *Simplified Chinese*, and *Traditional Chinese* for searching on Google Scholar.  *Japanese:*  目玉模様\|眼状紋 忌避\|捕食\|防除\|威嚇\|擬態 鳥\|鳴禽\|蝶\|蛾\|鱗翅目\|芋虫\|幼虫 実験\|研究  *Polish:*  oko\|oczy skrzydla\|wzor\|plama ochrona\|unikanie\|drapieżnik\|zapobieganie\|zastraszenie ptak\|motyl\|gasienica\|owad eksperyment\|badania  *Portuguese:*  ocelo\|“mancha ocelar”\|“olhos falsos”\|“falsos olhos” evitar\|predador\|prevenção\|intimidação ave\|pássaro\|borboleta\|mariposa\|lagarta experimento\|estudo  *Russian:*  глаз\|глазa избегать\|хищник\|профилактика\|запугивание птица\|бабочка\|мотылек\|Воробьинообразные\|Чешуекрылые\|Гусеница эксперимент\|изучать  *Spanish:*  ocelo\|“ojos falsos”\|”falsos ojos” evitar\|depredador\|prevención\|intimidación ave\|pájaro\|mariposa\|polilla\|oruga experimento\|estudio  *Simplified chinese:*  眼点 避免\|捕食者\|预防\|恐吓\|模仿 鸟类\|鸟\|雀\|蝴蝶\|蛾\|鳞翅目\|毛毛虫 实验\|试验\|学习  *Traditional chinese*:  眼點 避免\|捕食者\|預防\|恐嚇\|模仿 鳥類\|鳥\|雀\|蝴蝶\|蛾\|鱗翅目\|毛毛蟲 實驗\|試驗\|學習 |
