# Supplementary material for: A systematic review and meta-analysis of eyespot anti-predator mechanisms
Source: eLife. 2024 Dec 12;13:RP96338. doi: 10.7554/eLife.96338 (PMC11637465; doi:10.7554/eLife.96338)
Supplement: Supplementary file 5. — AM obtained the pictures from lepdata.org/photos/animals/ and https://data.nhm.ac.uk/ and measured the eyespot diameters. Raw data is available here: https://ayumi-495.github.io/eyespot/ and on GitHub (copy archived at Mizuno, 2024) and Zenodo. [file elife-96338-supp5.docx]

**Supplementary file 5**

| Median | Range |
| --- | --- |
| 3.41 | 1.82 – 5.04 |
